# *Cladomorphus petropolisensis*, a New Species of Stick Insect from the Atlantic Forest, Rio de Janeiro, Brazil †

**DOI:** 10.3390/ani12202871

**Published:** 2022-10-21

**Authors:** Jane Costa, Jacenir R. S. Mallet, Daniela Maeda Takiya

**Affiliations:** 1 Laboratório de Biodiversidade Entomológica, Instituto Oswaldo Cruz, Fiocruz, Rio de Janeiro 21040-360, Brazil; 2 Laboratório Interdisciplinar de Vigilância Entomológica em Diptera e Hemiptera, Instituto Oswaldo Cruz, Fiocruz, Rio de Janeiro 21040-361, Brazil; 3 Laboratório de Entomologia, Departamento de Zoologia, Instituto de Biologia, Universidade Federal do Rio de Janeiro, Rio de Janeiro 21941-901, Brazil

**Keywords:** Phasmatodea, stick insect, Petrópolis, mitochondrial COI gene

## Abstract

**Simple Summary:**

The order Phasmatodea includes the longest Brazilian insects, known by their remarkable morphological and behavioural adaptations for camouflage such as sticks, moss, and leaves; they are predominantly nocturnal and phytophagous insects. *Cladomorphus phyllinus* Gray, 1835 is one of the most common and best-known stick insect species in Brazil. It feeds mainly on guava leaves, angico, and powder-puff, and reproduces sexually and asexually. *Cladomorphus phyllinus* presents marked sexual dimorphism in the adult: winged males are significantly smaller in size than females and can reach up to 13 cm, while females can reach 23 cm in length and are apterous. A female specimen collected in the Atlantic Forest in Petrópolis, Rio de Janeiro, Brazil, was compared with a *C. phyllinus* specimen, identified according to published literature. The differences between the two specimens related to the general size and several morphological characters were observed. In order to add evidence that the recently collected specimen belonged to a species distinct from *C. phyllinus*, part of the cytochrome oxidase I (COI) gene was sequenced and analysed. The comparative analysis of the COI sequences from the two specimens revealed significant differences that, together with the morphological characters and recorded sympatry of the two specimens, support the existence of a new species, which is described here as *Cladomorphus petropolisensis.*

**Abstract:**

*Cladomorphus petropolisensis* sp. nov., a new species of stick insect from Petrópolis, Rio de Janeiro, Brazil, is herein described and compared to the other sympatric species, *C. phyllinus* Gray, 1835 (Phasmatidae, Cladomorphinae). The description of the new species is supported by morphological and molecular evidence. Kimura-2-parameter (K2P) intraspecific COI divergences among the holotype of *C. petropolisensis* sp. nov. and *C. phyllinus* individuals ranged from 2.9% to 4.4%, which are suggestive of distinct species, especially when considering that all *Cladomorphus* individuals studied were collected in the Petrópolis municipality. The new species can be distinguished from *C. phyllinus* Gray, 1835 by several characteristics: smaller size, the presence of two spines on the hind femora, the relative longer length of the ovipositor, and spiny tegument, especially in the mesonotum, sculpturing of the operculum of the egg.

## 1. Introduction

The Phasmatodea order includes the longest Brazilian insects, known by their remarkable morphological and behavioural adaptations for camouflage such as sticks, moss, and leaves; they are predominantly nocturnal and phytophagous insects [1]. The order encompasses 3423 species, of which 852 are recorded in the Neotropical region, with 230 occurring in Brazil [2,3]. The Neotropical phasmatodeans are poorly represented in phylogenetic studies as a result of the lack of basic scientific research on this group [4,5]. However, some Neotropical species were recently analysed by Simon et al. [5] and were mostly grouped in the large clade Occidophasmata Simon et al. 2019, while the Old World species were grouped in the clade Oriophasmata Simon et al. 2019.

In Brazil, phasmids are distributed throughout the country in distinct regions [6]; stick insects, as they are popularly known, generally show low reproductive capacity under optimal environmental conditions (low biotic potential), and a few records list some species as agricultural pests of economic importance [7].

The Phasmatodea is one of the least-studied insect orders in Brazil; recently, the state of the art of this group was reviewed by Madeira-Ott et al. [8]. New initiatives have been increasing the knowledge about this group, especially regarding taxonomy, morphology, biology, and ecology [9,10,11,12,13,14,15,16,17,18,19]. However, several factors bring difficulties to the study of this order in Brazil, such as intraspecific morphological variation, the low number of specimens collected, and a lack of specialised literature [20].

*Cladomorphus phyllinus* Gray, 1835 (Phasmatidae: Cladomorphinae: Cladomorphini) can be found in scientific collections, museums, schools, and universities for scientific and educational purposes, and is one of the most common and best-known stick insect species in Brazil. The species is polyphagous and feeds mainly on guava leaves *(Psidium guajava)*, angico *(Piptania* sp.), and powder-puff (*Calliandra* sp.), reproduces sexually and asexually by thelytokous parthenogenesis, and has nocturnal habits [21]. In the literature, some studies on *C. phyllinus* mainly present aspects of its biology, such as the papers of Alvarenga et al. [22] and Dorval et al. [23]. The authors analysed bred mated females fed on guava leaves and angico, respectively. The longevity, fertility, and viability of the eggs of parthenogenetic females of that species were also recently recorded [24].

The morphology of *C. phyllinus* is relatively well known [21,25], including a review of the subfamily [26]. Marked sexual dimorphism can be observed in the adult: winged males are significantly smaller in size than females and can reach up to 13 cm, while females can reach 23 cm in length and are apterous [21].

A female specimen recently collected in the Atlantic Forest in Petrópolis, Rio de Janeiro, Brazil, was kept in captivity and compared with a typical *C. phyllinus* female specimen, also collected from a nearby site. Differences were noticed between the two specimens mainly relating to the general size, eggs, ovipositor, number of spines on the hind femora, and on the spiny tegument of the mesonotum. In order to add evidence that the recently collected specimen belonged to a species distinct from typical *C. phyllinus*, part of the cytochrome oxidase I (COI) gene was sequenced and analysed. The comparative analysis of the COI sequences from the two specimens revealed significant differences that, together with morphological characters and recorded sympatry of the two specimens, support the existence of a new species of *Cladomorphus*, which is herein described.

## 2. Materials and Methods

### 2.1. Material Studied

Two adult female specimens of *Cladomorphus* were collected (5 January 2022 and 25 May 2022) in the city of Petrópolis, Rio de Janeiro, Brazil, in a fragment of Atlantic Forest, near an urbanised area of the city (22°30′18′′ S, 43°10′43′′ W). The collections were authorised by the Brazilian Ministry of the Environment (MMA), through the Biodiversity Authorization and Information System (SISBIO), process number 12123. The first collected specimen was identified as *C. phyllinus* according to the literature [2,21,25,26]. However, the second one did not fit the general characteristics of *C. phyllinus*. The two specimens were kept individually in captivity in order to obtain eggs. The plastic container used to rear the insects measured 60 × 40 × 40 cm, with translucent walls, which were perforated to ventilate the cage. The container was placed in an external area, protected by a roof, exposed to natural light cycles, but no direct sunlight. The temperature varied from 13° to 25 °C with an average of 24 °C and the relative humidity oscillated from 65% to 85% with an average of 75%. Both specimens were fed on leaves of guava (*Psidium guajava* L.; Myrtaceae). The bases of the stems were maintained in a jar with water inside the cage, to keep the plants fresh and keep humidity in the cage. These two specimens were preserved and deposited at the Entomological Collection of Instituto Oswaldo Cruz (CEIOC), Fiocruz, Rio de Janeiro.

### 2.2. COI Amplification and Sequencing

Genomic DNA was extracted from a single middle leg of each specimen using the DNeasy Blood and Tissue Kit (Qiagen, Hilden, Germany). The amplification and sequencing of the barcode region of the cytochrome oxidase I gene was conducted using the universal LCO 1490 and HCO 2198 primers [27]. The PCR reactions contained 5 μL of Taq buffer (Promega, Madison, WI, USA), 3.5 μL MgCl_2_ (25 mM, Promega), 1 μL dNTP mix (20 mM, Promega), 0.5 μL of each primer at 10 mM (Invitrogen, Thermo Fisher Scientific, Waltham, MA, USA), 0.2 μL Go^®^Taq DNA polymerase enzyme (Promega), 1.0 μL genomic DNA extract, and DEPC-treated water to a total volume of 25 μL. The thermocycling profile consisted of 3 min of initial denaturation at 94 °C, followed by 35 cycles of 1 min at 94 °C, 1 min at 50 °C, and 2 min at 72 °C, with a final extension step of 7 min at 72 °C. Amplicons were verified under UV light after agarose gel electrophoresis with DNA stained with GelRedTM (Biotium, Fremont) and purified using ExoSAP-IT^®^ (Applied Biosystems, Waltham, USA). The DNA sequencing of both the forward and reverse direction strands was conducted by Macrogen (Seoul, Korea). Complementary strands were assembled using Geneious Prime 2022. Both sequences generated as part of this study were deposited in GenBank under accessions OP430976 (Cladomorphus petropolisensis sp. nov., ENT6334) and OP430977 (Cladomorphus phyllinus ENT6333).

### 2.3. Phylogenetic Analyses and K2P Divergences

The COI sequences generated herein were analysed together with the only eleven COI sequences of *C. phyllinus* available at GenBank generated by Madeira-Ott et al. [8]. In addition, COI sequences from the other nine representatives of the Phasmatidae, Pseudophasmatidae, Lonchodidae, and Diapheromeridae were also included as outgroups. A 22-taxa alignment of 618 bp of COI was obtained with Geneious Prime and translated into amino acids to check for the absence of stop codons in the alignment.

Maximum likelihood and Bayesian inference mixed-model analyses were run in IQ-TREE 2 [28] and MrBayes 3.2 [29]. An appropriate substitution model and partitioning scheme was selected with BIC in ModelFinder in IQ-TREE [30] in two distinct analyses, one implementing all models allowed in IQ-TREE and another constraining to only MrBayes models. The respective substitution models for each COI codon position selected were as follows: position 1: TN + F + G4 or GTR + F + G4; position 2: TN + F + I or HKY + F + I; and position 3: TN + F + R2 or HKY + F + G4. Bayesian inference was run with two independent analyses of four Markov chain Monte Carlo (MCMC) chains during 10 million generations and sampled every 1000 with a 25% burnin. The results showed the adequate convergence of independent analyses and parameter mixing based on the average standard deviation of split frequencies < 0.005, parameter Potential Scale Reduction Factor  =  1.00, and minimum ESS values  > 2000. Clade support was inferred through 1000 replicates of an approximate Likelihood Ratio Test with the nonparametric Shimodaira-Hasegawa correction (SH-aLRT) [31] and Ultrafast bootstrap (UFBoot) [32] for ML analysis and Bayesian posterior probabilities (PP) [33].

Although eleven COI sequences of *C. phyllinus* were available at GenBank, only five of those were over 200 bp. Therefore, only these were used to calculate the K2P pairwise divergences between the single *C. petropolisensis* sp. nov. and the six sequences of *C. phyllinus* with complete deletion of sites with missing data in MEGA X [34].

### 2.4. Morphological Study

Photos of the general aspect and morphological details of the live specimens and eggs were taken (by JC) using a Samsung Note 8 camera, Samsung Electronics, Suwon, South Korea, with an additional lens.

Amplified images of the ornamentation of the operculum of the eggs were obtained in a scanning electron microscope (Jeol JSM 6390LV, Akishima, Tokyo, Japan) at the Electron Microscopy Platform of Instituto Oswaldo Cruz, at 15 Kv. Five eggs of each specimen were mounted on metallic supports, using double-sided tape and coated with gold using an evaporation system known as “sputtering”, where gold is removed from a massive electrode by ion bombardment in a high vacuum [35], using Balzers apparatus, by Oerlikon, Langenthal, Switzerland. The images obtained were captured directly on the computer using the. SemAphore 5.2 program, by E.W. Dijkstra, Austin, TX, USA.

The comparison of the morphological characters of the eggs (length, width, and the micropylar plate, colour) was based on ten samples of each specimen. Additionally, 60 eggs of each specimen, randomly selected from different periods of their oviposition, were observed under a stereoscopic microscope to check for the homogeneity and stability of the operculum shape and ornamentation.

## 3. Results

### 3.1. COI Evidence

The K2P intraspecific COI divergences of *C. phyllinus* ranged up to 1.9%, while divergences among the holotype of *C. petropolisensis* sp. nov. and *C. phyllinus* individuals ranged from 2.9% to 4.4% (see Supplementary File S1). These latter high values among these individuals are suggestive of a distinct species, especially when considering that all *Cladomorphus* individuals were collected in the Petrópolis municipality.

**Figure 1:**
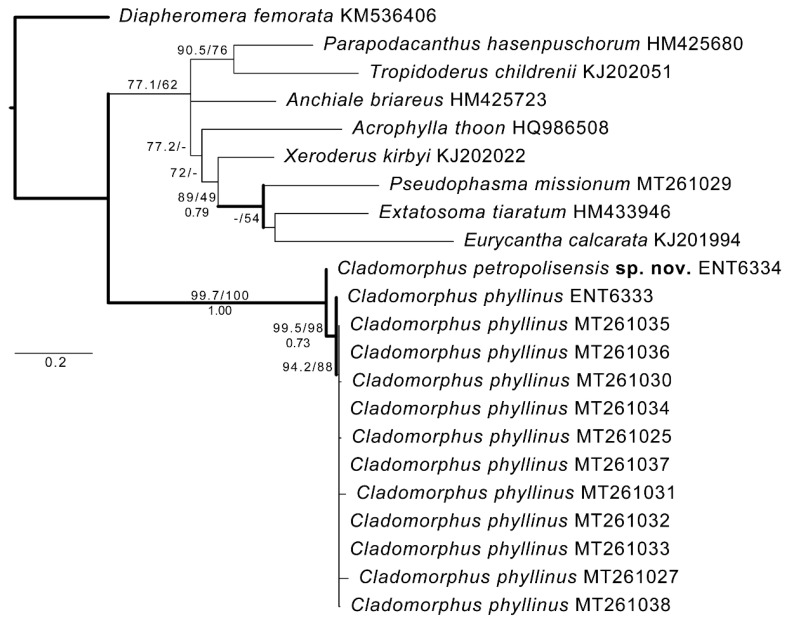
Maximum likelihood tree (−lnL = 2892.3608) of COI sequences (618 bp) of *Cladomorphus phyllinus* and *C. petropolisensis* sp. nov. Thicker branches were also recovered in Bayesian inference analysis. Clade support (over >50%) associated to nodes are: SH-aLRT/UFBoot above and BPP below.

The maximum likelihood analysis (−lnL = 2892.3608) and Bayesian inference (Figure 1) strongly suggested the placement of the proposed new species, *C. petropolisensis* sp. nov., as a sister to *C. phyllinus* with high clade support (SH-aLRT = 99.7, UFBoot = 100 and BPP = 1.00). All sequences of *C. phyllinus* were recovered as monophyletic and with moderate to high clade support as well (SH-aLRT = 99.5, UFBoot = 98, and BPP = 0.73).

### 3.2. Morphological Evidence

**Figure 2:**
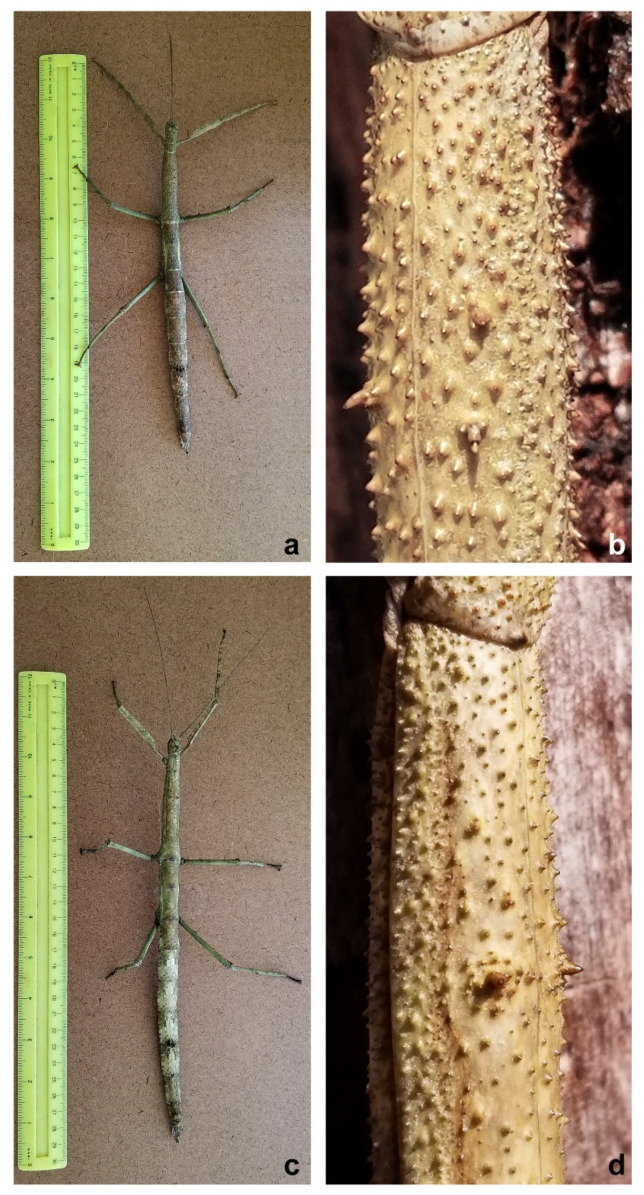
(**a**) General aspect of *Cladomorphus petropolisensis*; (**b**) detail of the tegument of the mesonotum showing three higher spines; (**c**) general aspect of *Cladomorphus phyllinus*; (**d**) detail of the mesonotum showing two higher spines.

The *Cladomorphus phyllinus* female specimen studied herein measured 25.2 cm in total length, while the holotype of the proposed new species measured 20.3 cm. there was a difference of 4.9 cm between the specimens (Figure 2).

**Figure 3:**
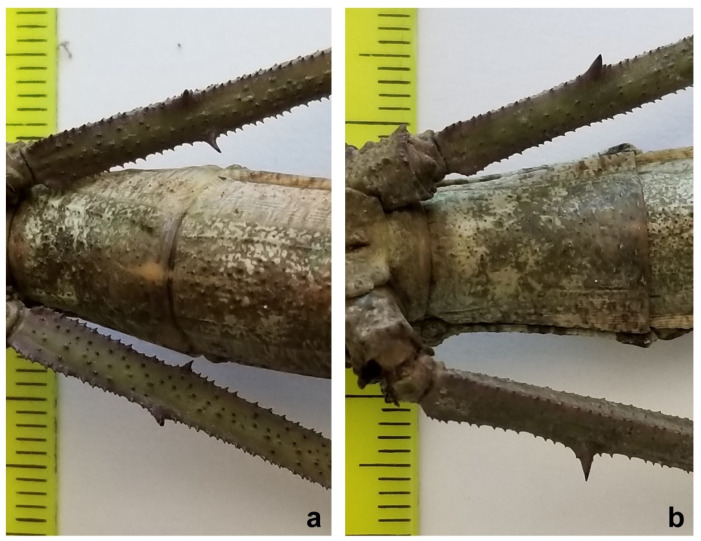
Femur of the hind legs: (**a**) *Cladomorphus petropolisensis* presenting two bigger spines; (**b**) *C. phyllinus* presenting one bigger spine.

**Figure 4:**
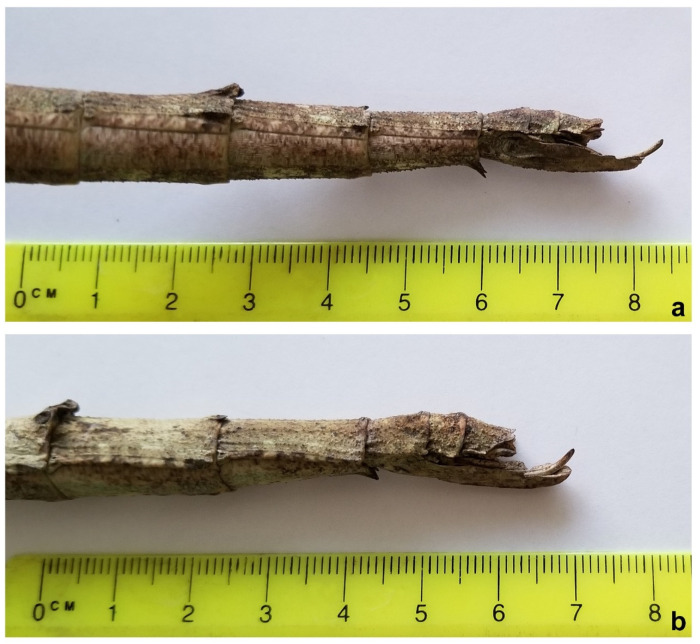
Ovipositor in lateral view. (**a**) *Cladomorphus petropolisensis*, measuring from the base of the subgenital plate (8th sternum) until the apex of the gonapophyses, is 24.5 mm, longer than the 7th + 8th tergum segments (21 mm); (**b**) *C. phyllinus* presenting a relative shorter subgenital plate and gonapophyses (25.3 mm), slightly shorter than the 7th + 8th tergum segments (28 mm).

Besides having a much smaller size than *C. phyllinus* [21,25,26], the tegument of the mesonotum of the holotype of the proposed new species is spinier than that observed for *C. phyllinus*, with three more prominent spines instead of two (Figure 2b,d). In addition, two spines were also observed on the femora of the hind legs instead of one as described in *C. phyllinus* (Figure 3), and its ovipositor is relatively longer than the one observed for *C. phyllinus* (Figure 4).

**Figure 5:**
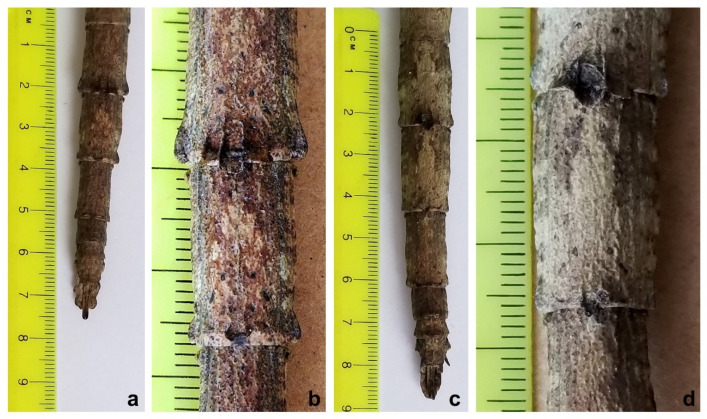
General aspect of the last abdominal segments and details of the fourth and fifth segments. (**a**,**b**) *Cladomorphus petropolisensis*; (**c**,**d**) *C. phyllinus*.

In the abdomen, the specimen collected more recently presents discrete expansions in the posterior limit of the IV and V abdominal segments, in the junction of the notum with the pleura, visible mainly in the dorsal view, which are absent in *C. phyllinus* (Figure 5).

**Figure 6:**
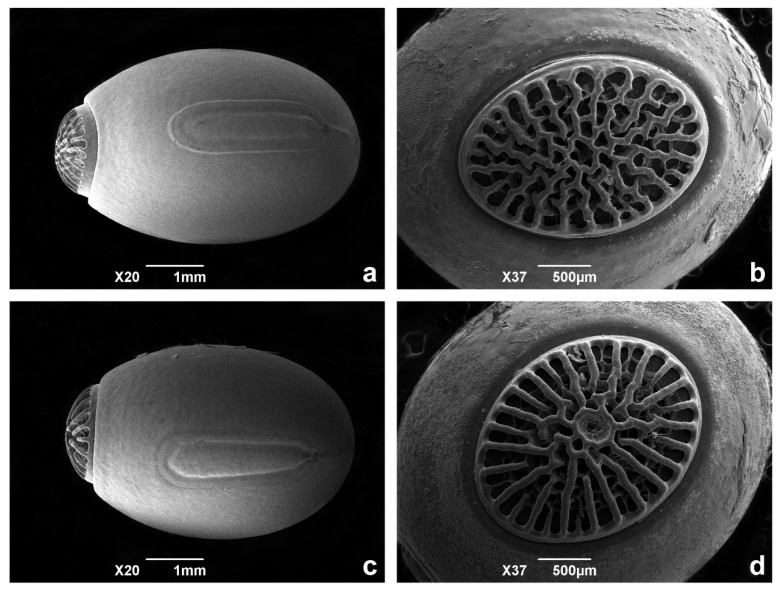
General aspect of the eggs under scanning electron microscopy. (**a**,**b**) *Cladomorphus petropolisensis*; (**c**,**d**) *C. phyllinus*.

**Figure 7:**
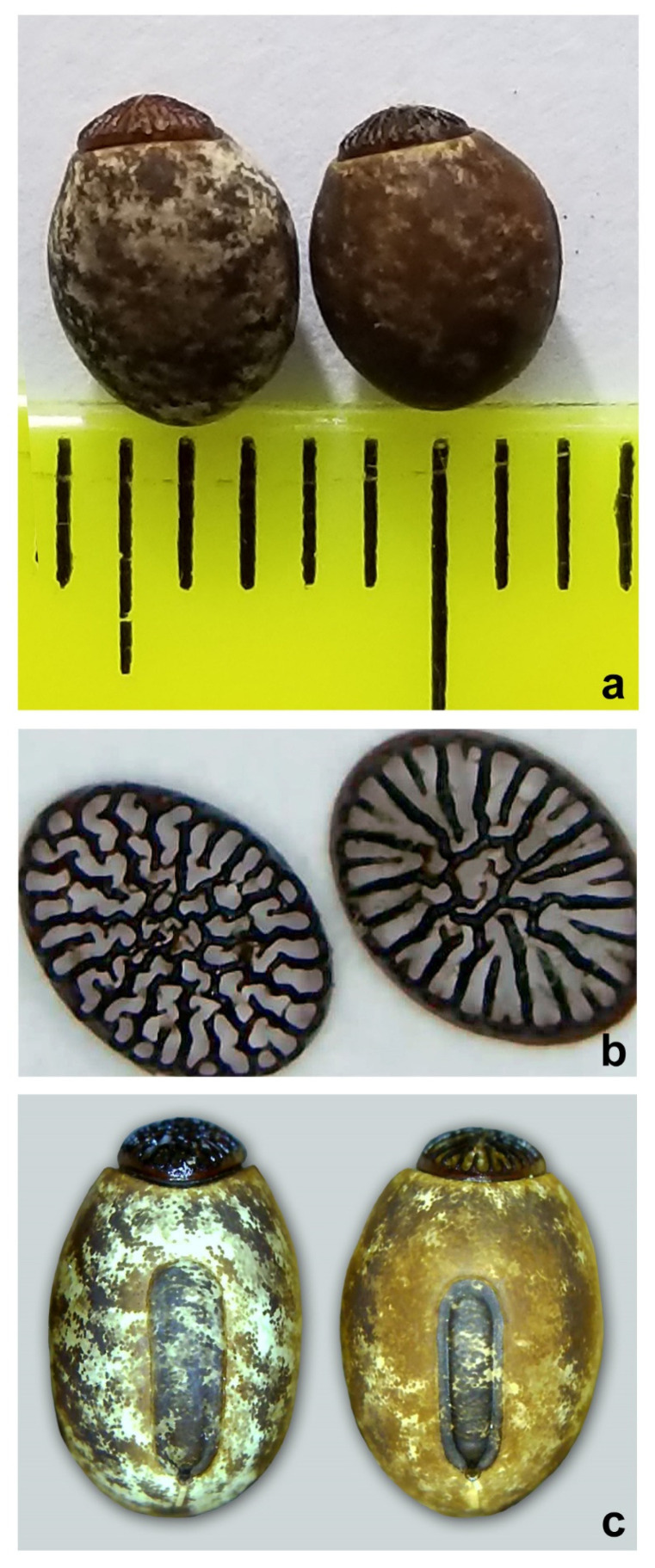
General aspect of the eggs under stereo microscopy. (**a**) *Cladomorphus petropolisensis* and *C. phyllinus*, respectively; (**b**) detail of the operculum for the mentioned species; (**c**) detail of the micropylar plate for the mentioned species.

The eggs of both specimens were observed under scanning electron microscopy (SEM) and stereomicroscopy. The pigmentation of the eggs is quite different as well: *C. phyllinus* presents a more uniform brown colour, while the other specimen is irregularly coloured in beige, light brown, and dark brown. The ornamentation of the operculum is distinct for the two specimens. The micropylar plate is also different, as the *C. phyllinus* is narrower and presents a darker colour that is very distinct from the rest of the egg, while in the other specimen, it is always very irregularly pigmentated, as for the rest of the egg (Figure 6 and Figure 7).

### 3.3. Taxonomy

Considering the differences in morphological characters mentioned above and the phylogenetic position and divergences of the COI obtained for *C. phyllinus* and the other specimen, a description of the new species, *Cladomorphus petropolisensis* sp. nov., is provided below.

*Cladomorphus petropolisensis* sp. nov. (Figure 2, Figure 3, Figure 4, Figure 5, Figure 6 and Figure 7)

Type locality: Petrópolis, Rio de Janeiro State, Brazil.

**Figure 8:**
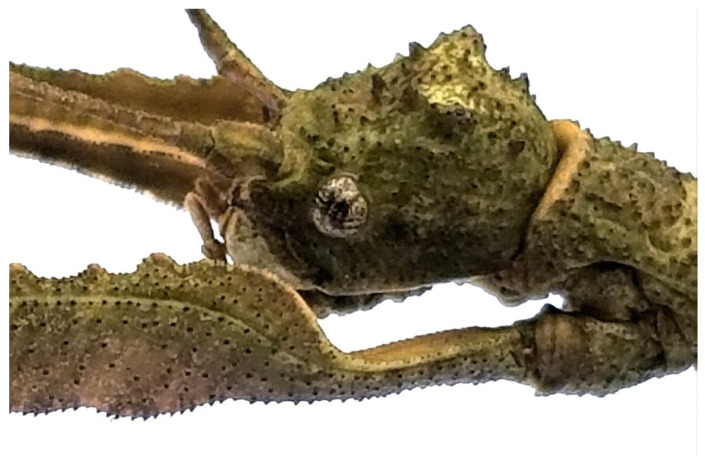
Head in lateral view, total length 9 mm, showing the vertex with prominent tubercles and details of the femur.

Description of female holotype. Apterous, body: robust, texture: rough, colour: light brown, clear in the posterior intersegment lines of the thorax and in the pleura of the mesonotum, darker areas in the distal part of the fourth abdominal segment and in the last three segments. Body: length 203 mm and width 12 mm at metanotum; head: width 0.88 of the length (width 8 mm in dorsal view, length 9 mm in lateral view); filiform antennae with 45 segments, dark brown, uniformly coloured from base to apex. Vertex with two symmetrical tubercles (Figure 8). Thorax: prothorax width 0.7 of the length (7/10 mm); mesothorax width 0.25 of length (10/40 mm), and metathorax width 0.41 of the length (12/29 mm). Legs: femora I, II, and III with length 34, 32, and 37 mm, respectively; femora I showing lightly spiny and undulating upper border and the inferior one very spiny (Figure 8); femora II and III each with two spines on ventral side (Figure 3); tibiae I, II, and III with lengths of 32, 28, and 35 mm, respectively; tibiae II and III exhibit tiny spines all over their length; basal tarsi as long as the next three and with a projection on the dorsal side. Abdomen: tergum IV with raised and gently undulating projection on distal margin; tergum IV and V with two lateral projections on the posterior border of the segment, in the lateral limit of the tergum; tergum V with reduced projection (Figure 5); preopercular organ in the sternum VI with three distinct spines on distal part, 3 mm long. Ovipositor in lateral view, showing the gonapophysis 4 mm longer than the VIII sternum. Cerci very short, each measuring 1.5 mm long (Figure 4).

Egg: capsule with smooth textured surface, with irregular spots in different colours from beige, to light brown, to dark brown. Total average length 5.14 mm (minimum 5 and maximum 5.5 mm), average width 4.15 mm (minimum 4 and maximum 4.5 mm). Operculum coloured reddish brown or darker with irregular reticules, converging to centre, frequently presenting small bifurcations, sometimes forming irregular cells (Figure 6 and Figure 7). Micropylar plate presenting a tubular shape, lightly displaced to the bottom of the egg, with the anterior margin distant to the margin of the operculum and measuring 0.65–0.70 of the total length of the egg (Figure 6).

Diagnostic characters. Shorter size, two spines on hind femur, posterior border of the IV abdominal segment with light lateral projections; relatively longer ovipositor, conspicuous spiny tegument of the mesothorax with three prominent spines on mesonotum, ornamentation of the operculum of the egg.

DNA sequences. The COI sequences generated herein from the holotype are available at GenBank (accession number OP430976).

Etymology. The name is allusive to the city of Petrópolis where the specimen was collected.

Type material. Holotype, female, Brazil, Rio de Janeiro, Petrópolis, 25 May 2022, Jane Costa col, CEIOC, DNA voucher ENT6334.

### 3.4. Additional Comparative Material

*Cladomorphus phyllinus.* One female, Brazil, Rio de Janeiro, Petrópolis, 5 January 2022, Jane Costa col, CEIOC, DNA voucher ENT6333. COI sequence GenBank accession number OP430977.

## 4. Discussion

In Phasmatodea, cases of remarkable sexual dimorphism and a degree of polymorphism are quite frequent, bringing difficulties for taxonomic studies. Nevertheless, molecular approaches have been of great importance to delimit distinct independent evolutionary unities, not just in that group, but also in several insect orders [36,37,38,39]. DNA sequences have been commonly and recently used to recover the higher-level phylogenetic relationships of phasmids [5,40,41,42,43,44,45]. However, it has been more scarcely used in an integrative taxonomy context to help species delimitation [46,47]. Herein, analyses of COI sequences were applied to support species distinctions. The results found are compatible with the existence of two sympatric species in Petrópolis, Rio de Janeiro, Brazil: *C. phyllinus* and *C. petropolisensis* sp. nov. Although there is little information on COI divergences among stick insects, the K2P divergences found between the holotype of the proposed new species and of sympatric *C. phyllinus* individuals (2.9–4.4%) are comparable to the interspecific divergences found from sister species in the literature. For example, the recently described *Achrioptera manga* Glaw et al., 2019 differs by 4.4% COI divergence to its sister species *A. fallax* Coquerel, 1861 and *Glawiana glawi* Hennemann and Conle, 2004 differs in 3.8% from *A. spinosissima* (Kirby, 1891) [47].

Due to the sexual dimorphism frequently observed in this group of insects, several species were described based on the morphology of just one specimen, and later, some of them were placed in synonymy [48,49]. Herein, the species description of *C. petropolisensis* sp. nov. is based on various female morphological characters, stereo and scanning electron microscopy of the eggs, the geographic distribution, and sequences of the mitochondrial COI gene. All of them lend support to the existence of a new species distinct from *C. phyllinus*. The characteristics observed for the *C. phyllinus* specimen matches with the ones already described in the literature by distinct authors [2,21,25,26]. Nevertheless, previous authors mentioned the intraspecific variation observed in the colour pattern of this species. However, the general characteristics already described in the literature for *C. phyllinus* such as general size, eggs, one spine on the hind femur, and the relative length of the ovipositor enabled the identification of all specimens as *C. phyllinus*.

Currently, according to Brock et al. [2], *Cladomorphus* includes six valid species recorded from Brazil: *C. ceratocephalus* Gray, 1835, *C. marcelloi* (Piza, 1974), *C. michaelis* (Redtenbacher, 1908), *C. phyllinus, C. rubus* (Saussure 1861), and *C. trimariensis* Kumagai and Fonseca, 2009. Comparatively analysing the mentioned species, *C. petropolisensis* is most similar to *C. phyllinus* and morphologically distinct from the others. In addition, two of the species are only recorded in Bahia State (*C. rubus, C. michaelis*), much further north from Rio de Janeiro, Brazil, and *C. ceretocephalus* and *C. trimariensis* are also remarkably distinct in terms of morphology.

The specimens of *C. phyllinus* and *C. petropolisensis* sp. nov. herein studied were collected at the adult stage and, therefore, the significant differences in the general size recorded cannot be correlated to artificial conditions in captivity. A difference in length of almost 5 cm (4.9 cm) was observed between the two specimens, both measured on the day of capture.

The morphological feature of the eggs is considered by Clark [50,51] as an important diagnostic character for phasmatodeans. The author stressed that not just species, but also genera can be identified by the egg structure. The two species presented stability, homogeneity, and consistent differences in egg morphology, attesting to the relevance of this character for identifying the studied species. The SEM of the eggs illustrated the morphological distinctions in ornamentations of the operculum and micropylar plate of the eggs of both species. Interestingly, despite the great difference in the general size observed between *C. phyllinus* and *C. petropolisensis*, their eggs did not show significant differences in size. It is important to mention that the new ornamentation of the eggs was not found in the literature for any other *Cladomorphus* species, being therefore a new morphological pattern for this genus.

## 5. Conclusions

*Cladomorphus petropolisensis* sp. nov. is phylogenetically related to *C. phyllinus* and can be easily distinguished by the shorter size of the apterous female, the number of spines on the hind femora, the relatively longer ovipositor and discreet expansions in the IV and V tergum segments, and the ornamentation of the operculum of the egg. Both species are found in Petrópolis city, amidst the Atlantic Forest and, interestingly, the holotype of *C. petropolisensis* sp. nov. was collected just 8 km away from where *C. phyllinus* is known to occur, reinforcing the morphological and genetic differences among them. This study brought unprecedented morphological and molecular information to the *Cladomorphus* genus, allowing the taxonomy of the new species *C. petropolisensis* sp. nov. to be addressed. This finding opens up new avenues to be explored in future studies, such as, for example, the reproductive compatibility among them, their phenotypic variability, and the cytogenetics, which are now being prepared, among other approaches.

## Data Availability

The insects used in this study are deposited in the Entomological Collection of Instituto Oswaldo Cruz (CEIOC), Fiocruz, Rio de Janeiro. The DNA sequences generated herein are deposited at GenBank under accession numbers OP430976 and OP430977.

## Supplementary Materials

The following supporting information can be downloaded at: https://www.mdpi.com/article/10.3390/ani12202871/s1, File S1: Divergences among the holotype of *C. petropolisensis* sp. nov. and *C. phyllinus* individuals.
